# EMPIRE: a highly parallel semiempirical molecular orbital program: 2: periodic boundary conditions

**DOI:** 10.1007/s00894-015-2692-3

**Published:** 2015-05-17

**Authors:** Johannes T. Margraf, Matthias Hennemann, Bernd Meyer, Timothy Clark

**Affiliations:** Computer-Chemie-Centrum and Interdisciplinary Center for Molecular Materials, Friedrich-Alexander-Universität Erlangen-Nürnberg, Nägelsbachstraße 25, 91052 Erlangen, Germany

## Abstract

ᅟ

**Electronic supplementary material:**

The online version of this article (doi:10.1007/s00894-015-2692-3) contains supplementary material, which is available to authorized users.

**Graphical Abstract:**
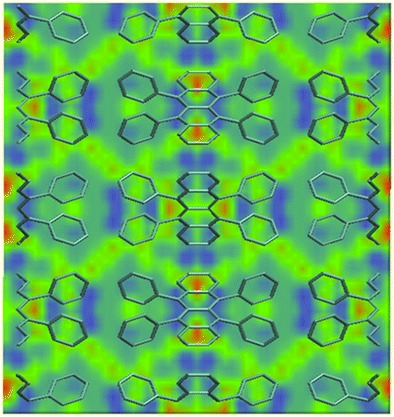
ᅟ

## Introduction

The increasing role of quantum chemical calculations in drug and materials design has led to a demand for methods that can describe the electronic structures of large and complex systems. Semiempirical methods based on the neglect of diatomic differential overlap (NDDO) approximation (e.g., the MNDO [1, 2], MNDO/d [3], AM1 [4], AM1* [5], and PMx [6–8] methods) are important representatives of such approaches. Many of these methods have been implemented in the massively parallel program EMPIRE [9], which makes the full quantum-mechanical treatment of systems containing 100,000 atoms or more possible.

Periodic boundary conditions (PBC) enable quantum chemical programs to treat condensed-phase systems, such as proteins in a periodic water box or solids. This allows molecular materials to be studied in their “native” environment, instead of comparing experimental bulk properties with gas-phase monomer calculations. For semiempirical methods, the most practical way of implementing PBC is the cyclic-cluster approach [10–12] in which the system is approximated by a supercell and by imposing Born–von Karman boundary conditions [13]. Using a large unit cell allows the calculation to be performed entirely in real space. This is easily affordable because of the generally low computational cost of NDDO calculations. The main advantage of this technique is that program features like the calculation of local properties [14] or excited states are directly transferable from nonperiodic calculations [15]. We have, for example, used periodic EMPIRE calculations to model amorphous carbon [16].

EMPIRE, which was especially designed for calculations on systems with very many atoms, is also suitable for use on systems with very large unit cells (e.g., disordered and amorphous systems). EMPIRE can, for example, be used in combination with a classical molecular dynamics (MD) code to perform electronic structure calculations on snapshots from an MD run on a periodic system. In the first section of this paper, we discuss the implementation of periodic boundary conditions in EMPIRE. In the second, the program performance is discussed briefly. Finally, some exemplary applications of large-scale periodic NDDO calculations are shown.

## Implementation

Periodic calculations in EMPIRE are performed entirely in real space. Therefore, no major changes to the NDDO SCF algorithm were required. Only small adjustments are necessary in the treatment of two-electron two-center integrals: the exchange energy and electrostatic interactions. These adjustments will be discussed below. For more background information, we refer the reader to [10, 12].

### Two-electron two-center integrals

The values of the two-electron two-center integrals* γ*_*AB*_ used in NDDO calculations (and the associated potential) quickly decrease with the distance between the centers but remain nonzero. Since these small values add up to unphysical, infinite potentials in a periodic system, they must be corrected [12]. This is achieved by introducing a Gaussian damping function that sets in at a cutoff value *c*_cut_ (the default is 10.0 bohrs). The functional form for these integrals is (in atomic units)

1 $$ {\gamma}_{AB}=\frac{1}{r+{{\mathrm{e}}^{-0.25{\left(r-{c}_{\mathrm{cut}}\right)}^2\left(\frac{1}{G_A}+\frac{1}{G_B}\right)}}^2},\kern0.5em r>{c}_{\mathrm{cut}}, $$

where *G*_*A*_ and *G*_*B*_ are parameterized constants for elements *A* and *B*, respectively, and *r* is the distance between the two centers.

### Exchange interaction energy

The next adjustment is required with respect to the two-electron two-center exchange integrals *h*_*μν*_, which appear in the Fock matrix. These terms depend on the density matrix elements *P*_*μν*_. In a periodic calculation, the exchange interactions for an orbital centered on a given atom are only evaluated within the Wigner–Seitz cell surrounding it. The net result is the neglect of very weak exchange interactions with distant electrons, which causes no loss in accuracy [10].

### Electrostatic interactions

MNDO-like NDDO methods describe electrostatic electron–electron and electron–core interactions using multipole–multipole interactions. In a periodic system, small interactions with an infinite number of distant charges lead to unphysical results. This can be alleviated by introducing a simple, one-parameter screening function.

Simply put, distant charges are relocated to an effective distance *r*_eff_, which is a function of the actual distance *r*. The space around a charge is divided into three regions, delimited by a parameter* α*: at close distances (*r* < *α*), the actual and effective distances are equal (*r*_eff_ =  *r*). At large distances (*r* > 2*α*), all charges are moved to a constant radius of 1.5*α*. In this manner, their effects cancel each other out due to symmetry [12]. In the intermediate region, the distance is scaled so as to satisfy the conditions

2 $$ {r}_{\mathrm{eff}}\left(\alpha \right)=r $$

and

3 $$ {r}_{\mathrm{eff}}\left(2\alpha \right)=1.5\alpha . $$

This scaling function [12] is defined as

4 $$ {r}_{\mathrm{eff}}=-\frac{\alpha }{2}+2r-\frac{r^2}{2\alpha }. $$

MNDO-like methods treat charges as distributed multipoles, i.e., point charges at a defined distance to a center. To keep this screening scheme completely consistent, the positions of all individual point charges that make up distant multipoles would need to be scaled. This is undesirable because it would require calculating the distance between all atoms and all distributed multipole point charges. To avoid this, we introduce a second scaling function for the distance between the point charges of a multipole and the atom on which they are centered. At distances* r* < *α*, the multipoles are unaffected. At large distances* r* > 2*α*, all multipoles are reduced to point charges. In the intermediate region* α* <* r * < 2*α*, the multipoles are scaled by a factor* λ*(*r*), with the boundary conditions

5 $$ \lambda \left(\alpha \right)=1 $$

and

6 $$ \lambda \left(2\alpha \right)=0. $$

The corresponding function is the derivative of (4):

7 $$ \lambda (r)=2-\frac{r}{\alpha }. $$

The effect of scaling the multipole size can be evaluated by considering three different scenarios for an interaction between a point-charge and a dipole. The rigorous but unpractical solution is to scale the positions of the constituent point charges of the dipole individually, whereas the simplest approach would be to scale only the center of the dipole. Finally, we can scale the center of the dipole according to (4) and the distances of the distributed point charges to their center by (7).

**Fig. 1:**
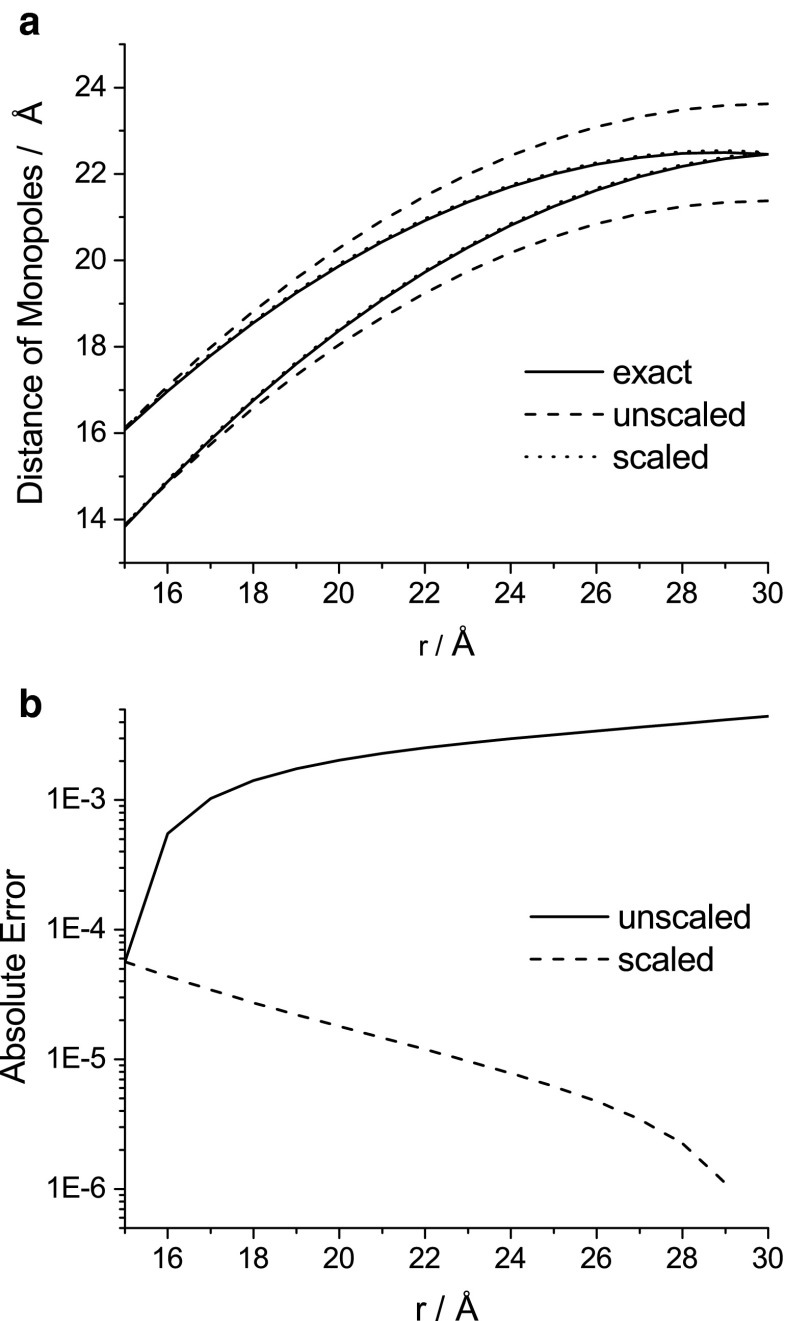
*Top*: positions of distributed multipole point charges in the exact, scaled, and unscaled scenarios.* Bottom*: absolute error in the Coulomb energy for the scaled and unscaled cases

Figure 1 shows the positions of the distributed monopoles for all three cases (*α* = 15.0 Å). Clearly, the scaling function* λ* is a practical way to describe the exact scaling of the multipole distance. This can also be shown by considering the Coulomb energy for the interaction between point charge and dipole. The dependence of the absolute error (i.e., the difference between the unscaled/scaled and exact cases) for this energy on the distance is also shown in Fig. 1. Below the cutoff value, all models are by definition equivalent. At the cutoff value, the scaled and unscaled errors are identical. At increasing distances, however, the error decreases for the scaled case and increases for the unscaled case.

## Performance

### Setting up periodic calculations

**Fig. 2:**
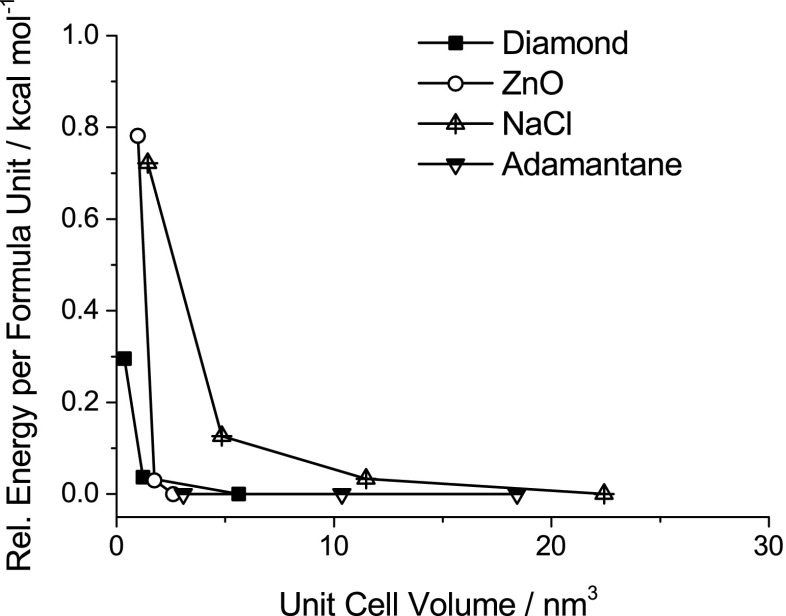
Energy convergence with respect to the unit cell volume for diamond, ZnO, NaCl, and adamantane

Periodic EMPIRE calculations require little more input than nonperiodic ones. Apart from the Cartesian coordinates of all atoms, a unit-cell vector is required for each periodic direction. Calculations can be periodic in one, two, or three dimensions. Since no k-space sampling is performed, the unit cell should be sufficiently large. The MOPAC manual suggests that 7–8 Å per repeat unit vector should be sufficient for most compounds, and larger unit cells should be used for highly conjugated π systems and small band-gap materials [17]. It is good practice to check the convergence of the calculated results with the size of the unit cell. Figure 2 shows the convergence of the calculated heat of formation with the unit-cell volume for diamond, ZnO, NaCl, and the adamantane molecular crystal. The convergence of the unit-cell size may differ for other properties, as Bredow et al. showed for excitation energies, where significantly larger cells were required than for the ground-state energy [15].

The electrostatic screening parameter can be modified via the keyword ScreeningR, which sets the value of 2*α* in Å. The default is 30.0 Å. This conservative cutoff corresponds to the MOPAC default. Lower cutoffs result in lower computational cost, but whether the heat of formation is affected by the change should be checked. The energy convergences and computational costs of different values of* α* are shown for diamond and ZnO in the “Electronic supplementary material” (ESM, Figs. S1 and S2).

### Single-node open MP scaling

**Fig. 3:**
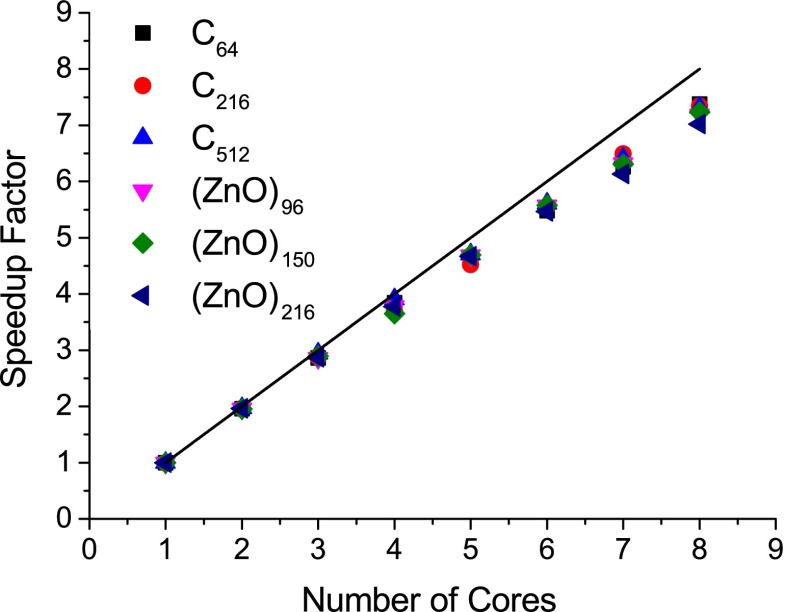
Relative speedup factors for OMP parallel calculations of differently sized diamond and ZnO unit cells, performed on a single node consisting of two quad-core 2.83-GHz Intel^®^ Xenon^®^ E5440 processors with 8 GB of memory. No hyperthreading was used

**Fig. 4:**
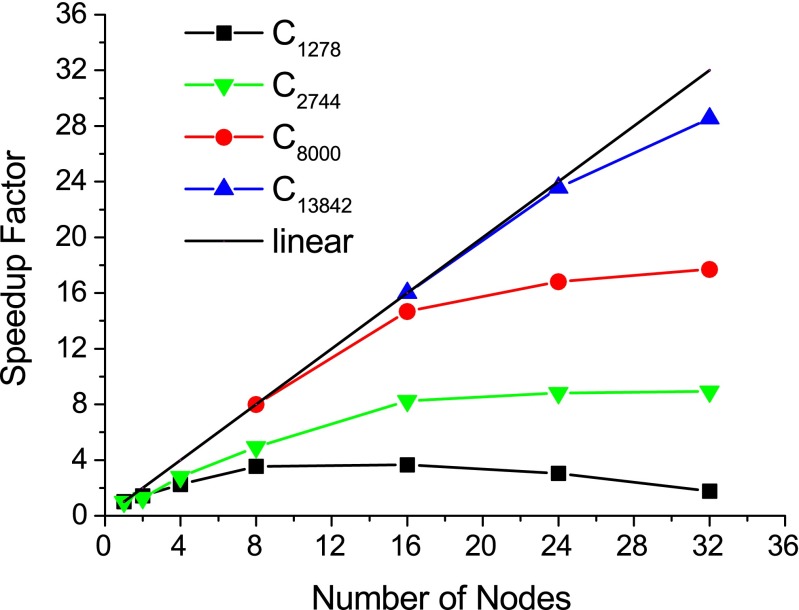
Relative speedup factors for MPI parallel calculations of differently sized diamond unit cells, performed on the LiMa cluster. Each node was equipped with two six-core Intel^®^ Xeon^®^ 5650 “Westmere” chips; the nodes were connected by an Infiniband interconnect fabric with 40 Gbit/s bandwith per link and direction. We used two MPI tasks per node and six OMP threads for each. No hyperthreading was used

Table 1 shows timings for AM1-SCF calculations of differently sized diamond and ZnO unit cells performed with the single-node OMP version of EMPIRE. The corresponding speedup for different numbers of cores is plotted in Fig. 3. The scaling is quite efficient; the speedup factor is >7 using eight cores for all systems investigated. The largest system considered here is the C_512_ unit cell, for which an SCF calculation takes less than 1 min on eight cores. This shows that on a modern desktop computer, periodic calculations with EMPIRE are absolutely affordable (see Table 2 and Fig. 4).

**Table 1:** Wall-clock times for AM1-SCF calculations performed with the single-node OMP version of EMPIRE. These calculations were performed on a node consisting of two quad-core 2.83-GHz Intel^®^ Xenon^®^ E5440 processors with 8 GB of memory. No hyperthreading was used

Unit cell	Number of orbitals	Wall-clock time (s) for* N* cores							
		*N* = 1	*N* = 2	*N* = 3	*N *= 4	*N* = 5	*N* = 6	*N* = 7	*N* = 8
C_64_	256	26.9	13.7	9.4	7.0	5.8	4.9	4.3	3.6
C_216_	864	111.4	56.7	38.7	30.1	24.6	20.0	17.2	15.2
C_512_	2048	428.3	217.7	145.7	109.6	91.1	76.4	66.8	58.6
(ZnO)_96_	768	69.9	35.7	24.4	18.5	14.9	12.5	11.1	9.7
(ZnO)_150_	1200	162.4	83.2	56.1	44.5	34.6	29.1	25.8	22.4
(ZnO)_216_	1728	224.0	114.2	77.9	59.4	48.0	41.0	36.5	31.9

**Table 2:** Wall-clock times for AM1 SCF calculations performed with the multi-node hybrid MPI/OMP version of EMPIRE. Each node was equipped with two six-core Intel^®^ Xeon^®^ 5650 “Westmere” chips; the nodes were connected by an Infiniband interconnect fabric with 40 Gbit/s bandwith per link and direction. We used two MPI tasks per node and six OMP threads for each. No hyperthreading was used

Unit cell	Number of orbitals	Wall-clock time (s) for* N* nodes				
		*N* = 4	*N* = 8	*N* = 16	*N* = 24	*N* = 32
C_1728_	6912	177.8	112.2	109.3	-	-
C_8000_	32000	-	5149.8	2806.1	2448.5	2327.7
C_13824_	55296	-	-	13813.6	9376.8	7741.4

### Multi-node hybrid OMP/MPI scaling

The scaling of the hybrid OMP/MPI multi-node version of EMPIRE was tested on the LiMa cluster at the Regionales Rechenzentrum Erlangen. Here, we used differently sized diamond unit cells from C_1,728_ to C_13,824_. Please note that very large unit cells also require large amounts of memory, especially because the integrals are stored, since their calculation is relatively expensive in periodic calculations. Therefore, it is not possible to use the same reference number of nodes when determining the scaling for these systems. The speedup is always relative to the lowest number of nodes feasible for a given system. Optimizing the SCF procedure for periodic calculations may improve the performance of EMPIRE on fewer nodes. As it is, the calculations scale very impressively up to twice the minimum number of nodes. Further increasing the number of nodes leads to a plateau.

## Application

The application of NDDO methods to crystalline materials has been thoroughly tested and evaluated by Stewart, and will therefore not be discussed here in any detail [18]. Instead, we would like to focus on two aspects unique to EMPIRE: firstly, the calculation of local properties; secondly, the fact that even unit cells with thousands of atoms can be treated easily.

### Local properties

A local property is any property that can be derived from the wavefunction of a structure and mapped onto a real-space grid, such as the electron density and the molecular electrostatic potential (MEP). These can be calculated with most electronic structure codes. EMPIRE (in combination with an auxiliary program) gives access to several additional local properties derived from molecular orbitals and their energies. These are the local electron affinity (EA_L_), ionization energy (IE_L_), electronegativity, and hardness, which have been used for biochemical QSPR studies and to predict the electron-transport properties of nanostructures [19–27].

**Fig. 5:**
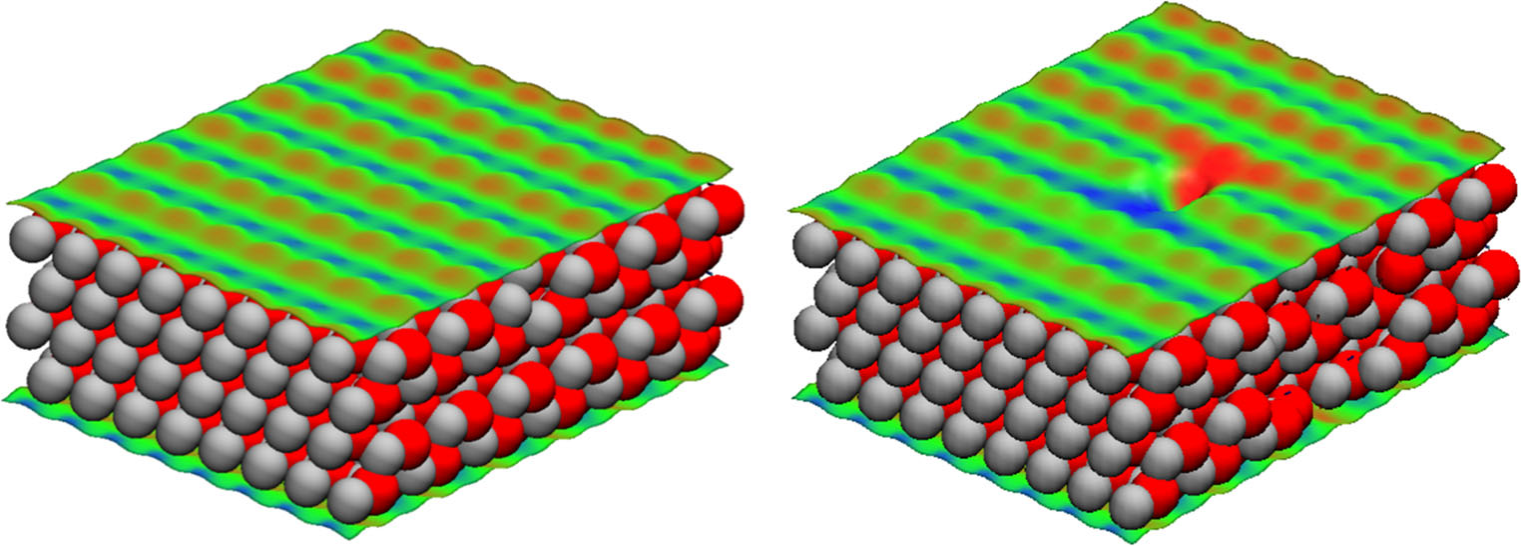
*Left*: the MNDO/d molecular electrostatic potential (MEP) projected onto an electron isodensity (0.01 e^−^ Å^−3^) surface of a ZnO$\left(10\overline{1}0\right)$ slab calculated with two-dimensional periodic boundary conditions. The* image on the right* shows the same slab after a ZnO dimer was removed from the surface. The surface color code ranges from −50.0 (*blue*) to 50.0 (*red*) kcal mol^−1^

Figure 5 shows the molecular electrostatic potentials of a pristine and a defective ZnO$\left(10\overline{1}0\right)$ surface. The nonpolar $\left(10\overline{1}0\right)$ surface consists of rows of ZnO dimers that are separated by trenches [28]. The most abundant atomic defects on this surface are ZnO dimer vacancies [29]. The entire geometry was re-optimized with the MNDO/d Hamiltonian for both the pristine and the defective surface. The removal of one ZnO dimer clearly affects the electrostatic potential around the defect, making it necessary to choose a large unit cell to avoid interactions of the defect with its periodic image. This calculation required around 1 min per optimization step and converged in 32 steps. The cell contains 766 atoms and 3,064 electrons. Note also that the MEP does not depend on simplifications such as point-charge models. The multipole formalism used in MNDO-like techniques gives a very good representation of the MEP calculated at higher levels of theory, including anisotropic distributions around heavy atoms [30].

**Fig. 6:**
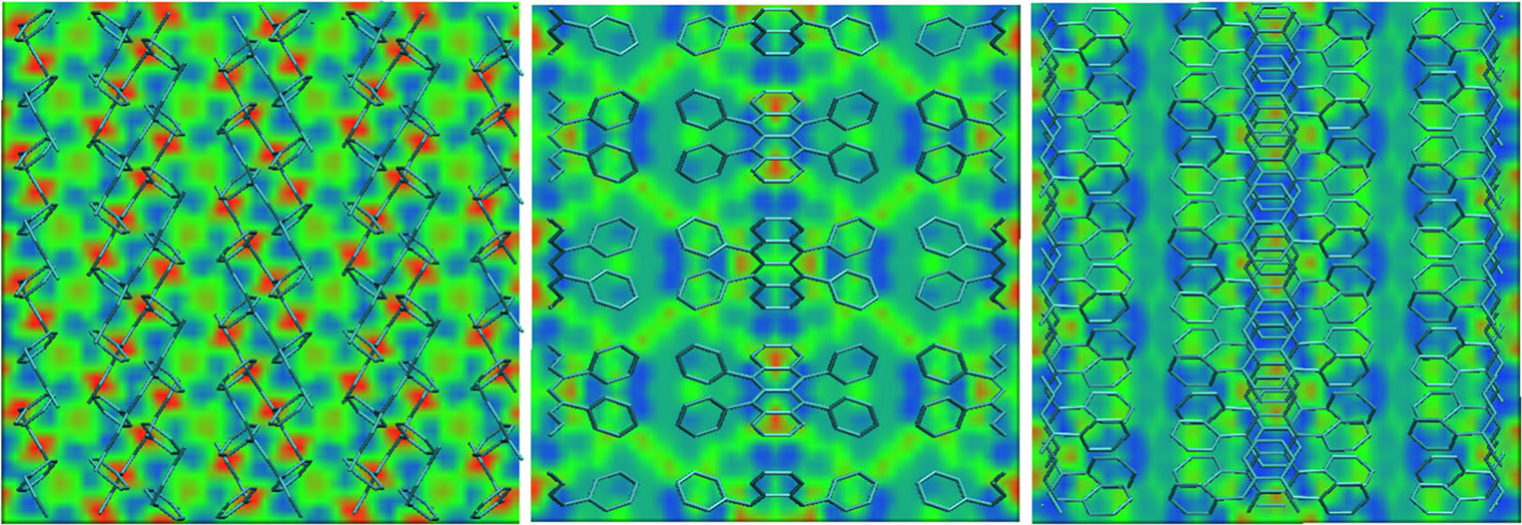
AM1 local ionization energy (IE_L_) volume slices cutting through the rubrene unit cell perpendicular to the* x*,* y*, and* z* directions. The color code ranges from 360 (*blue*) to 600 (*red*) kcal mol^−1^

A recent application of periodic local property maps lies in the study of charge transport in organic materials [25–27, 31]. In the condensed phase, the local ionization energy (IE_L_) and electron affinity (EA_L_) can be interpreted as the local valence-band maximum and conduction-band minimum, respectively. They can therefore be used to visualize the anisotropic electronic properties of a molecular crystal. More recently, the local properties have been used as external potentials to simulate charge transport (see [32]; Bauer T et al.,* A multi-agent quantum Monte Carlo model for charge transport: application to organic field-effect transistors*, submitted). Figure 6 shows the local ionization energy (IE_L_) of a rubrene crystal projected onto volume slices that cut through the unit cell along its main axes.

Low IE_L_ values (shown in blue) correspond to electron-donating/hole-conducting pathways, whereas high IE_L_ values (shown in red) represent energy barriers. In Fig. 6, the IE_L_ maps look vastly different depending on the orientation of the volume slice. This is in line with experimental reports, which show that the field-effect mobilities in rubrene single crystals depend strongly on the orientation of the contacts [33, 34].

### Large unit cells

**Fig. 7:**
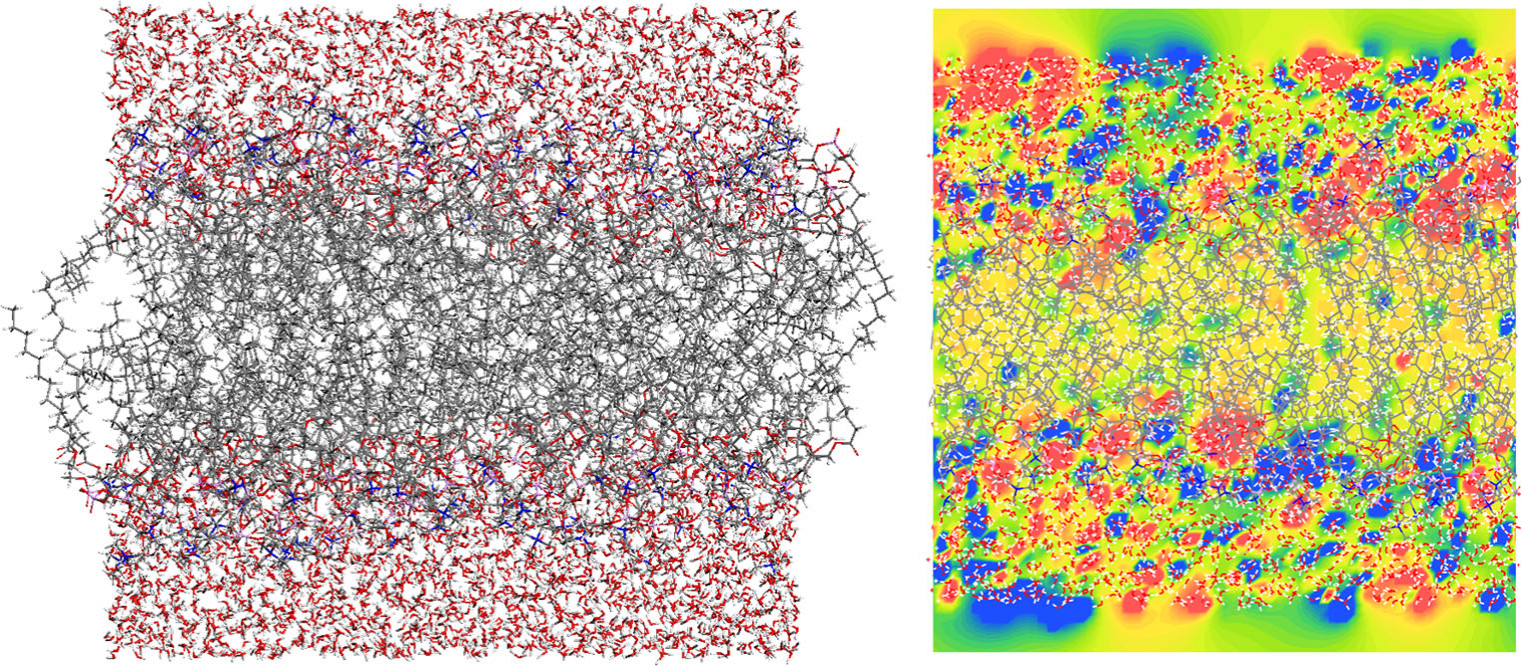
*Left*: model lipid bilayer membrane based on DLPC units and 3840 water molecules (taken from [34]).* Right*: MEP volume slice, color coded from −50 (*blue*) to 50 (*red*) kcal mol^−1^

As an example of a large system, we chose the solvated lipid bilayer membrane shown in Fig. 7. Specifically, the model consists of 128 1,2-dilauroyl-*sn*-glycero-3-phosphocholine (DLPC) and 3,840 water molecules equilibrated for 400 ns in a classical molecular dynamics simulation [35]. The unit cell contains 25,088 atoms and spans 62.502 × 65.506 × 58.441 Å^3^. An AM1-SCF calculation was performed on 384 cores of the LiMa cluster (64 MPI tasks on 32 nodes with 2 × 6 cores each). The SCF converged in 31 cycles and took a little over 3 h 7 min.

Note that periodic calculations of this size push double-precision (64-bit) arithmetic to its limit, since many small values are summed to a very large result during the energy summation. To avoid numerical inaccuracies for large systems, this step is performed in quadruple precision (128-bit), and special care is taken in the ordering of the summands.

The resulting HDF5 binary wavefunction file has a size of 21 GB and can be used to calculate local property maps. The molecular electrostatic potential across the membrane is shown in Fig. 7 (right). This clearly visualizes the polar water layer and head groups and the nonpolar lipid bilayer. Such calculations could, for instance, be used to predict the permeability of membranes to different chemicals.

**Fig. 8:**
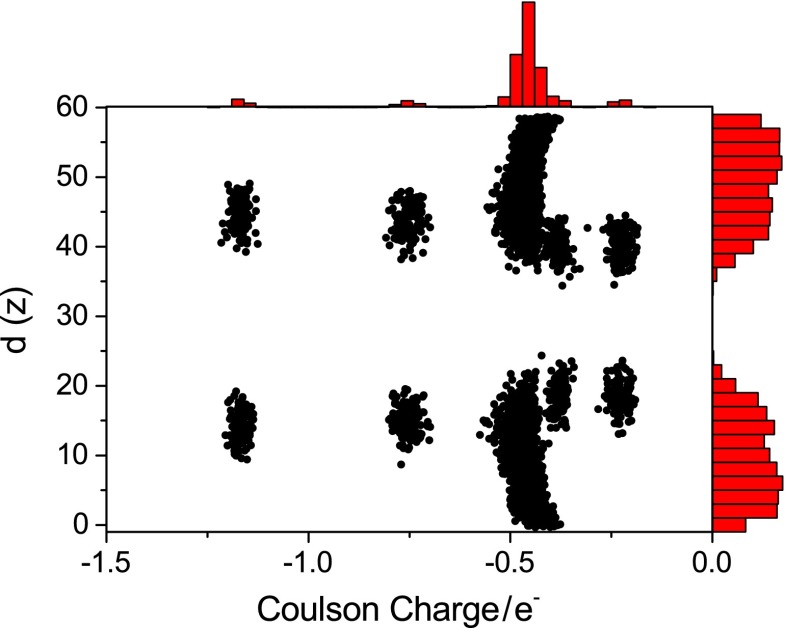
Distribution of Coulson charges and vertical coordinates of all oxygen atoms in the DLPC membrane/water system

Every EMPIRE calculation also includes a Coulson population analysis. Figure 8 shows a plot of the Coulson charges of all oxygen atoms as a function of their vertical position. In this plot, five charge groups are discernable, corresponding to the four chemically distinct oxygen atoms in DLPC and the oxygen atom in water. This presents an interesting perspective in the development of force fields for condensed-phase applications, since the charges can be derived directly for the solid or liquid of interest.

## Conclusions

We have implemented periodic boundary conditions in the massively parallel semiempirical molecular orbital theory code EMPIRE. The standard SCF procedure of EMPIRE reliably converges the wavefunctions of a broad range of periodic systems, including covalent, ionic, and molecular crystals and surfaces as well as disordered biological systems such as a lipid bilayer. Like the nonperiodic version of EMPIRE, the program is parallelized in the single-node version via open MP, and in the multi-node version via a hybrid open MP/MPI approach.

The single-node version was shown to perform well for calculations on unit cells containing between 64 and 512 atoms, and to scale very efficiently on up to eight cores. The multi-node version allows systems with tens of thousands of atoms to be treated; the largest system described here consisted of 25,088 atoms. The program scaling is similar to that observed for nonperiodic calculations with EMPIRE [9].

## Electronic supplementary material

ESM 1 (DOCX 90 kb)

## References

[1] Dewar MJS, Thiel W (1977) Ground states of molecules, 38. The MNDO method. Approximations and parameters. J Am Chem Soc 99:4899. doi:10.1021/ja00457a004

[2] Dewar MJS, Thiel W (1977) Ground states of molecules, 39. MNDO results for molecules containing hydrogen, carbon, nitrogen, and oxygen. J Am Chem Soc 99:4907. doi:10.1021/ja00457a005

[3] Thiel W, Voityuk A (1996) Extension of MNDO to* d*-orbitals—parameters and results for the second-row elements and for the zinc group. J Phys Chem 100:616. doi:10.1021/jp952148o

[4] Dewar MJS, Zoebisch EG, Healy EF, Stewart JJP (1985) AM1: a new general purpose quantum mechanical model. J Am Chem Soc 107:3902. doi:10.1021/ja00299a024

[5] Winget P, Horn AC, Selçuki C, Martin B, Clark T (2003) AM1* parameters for phosphorus, sulfur and chlorine. J Mol Model 9:408. doi:10.1007/s00894-003-0156-7 10.1007/s00894-003-0156-7 12955599

[6] Stewart JJP (1989) Optimization of parameters for semi-empirical methods I: method. J Comput Chem 10:209–220. doi:10.1002/jcc.540100208

[7] Stewart JJP (2007) Optimization of parameters for semiempirical methods V: modification of NDDO approximations and application to 70 elements. J Mol Model 13:1173. doi:10.1007/s00894-007-0233-4 10.1007/s00894-007-0233-4 PMC2039871 17828561

[8] Stewart JJP (2013) Optimization of parameters for semiempirical methods VI: more modifications to the NDDO approximations and re-optimization of parameters. J Mol Model 19:1–32. doi:10.1007/s00894-012-1667-x 10.1007/s00894-012-1667-x PMC3536963 23187683

[9] Hennemann M, Clark T (2014) EMPIRE: a highly parallel semiempirical molecular orbital program: 1: self-consistent field calculations. J Mol Model 20:2331. doi:10.1007/s00894-014-2331-4 10.1007/s00894-014-2331-4 24944094

[10] Perkins PG, Stewart JJP (1980) Cluster model for solids. J Chem Soc Faraday Trans 2 76:520. doi: 10.1039/F29807600520

[11] Bredow T, Geudtner G, Jug K (2001) Development of the cyclic cluster approach for ionic systems. J Comput Chem 22:89–101. doi:10.1002/1096-987X(20010115)22:1%3C89::AID-JCC9%3E3.0.CO;2-7

[12] Stewart JJP (2000). A practical method for modeling solids using semiempirical methods. J Mol Struct.

[13] Born M, Kármán T (1912). Über Schwingungen in Raumgittern. Phys Z.

[14] Ehresmann B, Martin B, Horn AHC, Clark T (2003). Local molecular properties and their use in predicting reactivity. J Mol Model.

[15] Gadaczek I, Hintze KJ, Bredow T (2012) Periodic calculations of excited states for solids using a semiempirical approach. Phys Chem Chem Phys 14:741–750. doi:10.1039/c1cp22871d 10.1039/c1cp22871d 22117222

[16] Margraf JT, Strauss V, Guldi DM, Clark T (2015) The electronic structure of amorphous carbon nanodots. J Phys Chem B 119:ASAP. doi:10.1021/jp510620j 10.1021/jp510620j 25731776

[17] Stewart JJP (2015) MOPAC online manual. Stewart Computational Chemistry, Colorado Springs. http://openmopac.net/manual/Solids_cluster.html. Accessed 5 April 2015

[18] Stewart JJP (2008). Application of the PM6 method to modeling the solid state. J Mol Model.

[19] Ehresmann B, de Groot MJ, Alex A, Clark T (2004) New molecular descriptors based on local properties at the molecular surface and a boiling-point model derived from them. J Chem Inf Comput Sci 44:658–668. doi:10.1021/ci034215e 10.1021/ci034215e 15032548

[20] Güssregen A, Matter H, Hessler G, Müller M, Schmidt F, Clark T (2012) 3D-QSAR based on quantum-chemical molecular fields: towards an improved description of halogen interactions. J Chem Inf Model 52:2441–2453. doi:10.1021/ci300253z 10.1021/ci300253z 22917472

[21] Ehresmann B, de Groot MJ, Clark T (2005) Surface-integral QSPR models: local energy properties. J Chem Inf Model 45:1053–1060. doi:10.1021/ci050025n 10.1021/ci050025n 16045301

[22] Clark T (2010) The local electron affinity for non-minimal basis sets. J Mol Model 16:1231–1238. doi:10.1007/s00894-009-0607-x 10.1007/s00894-009-0607-x 20063173

[23] ElKerdawy A, Wick CR, Hennemann M, Clark T (2012) Predicting the sites and energies of noncovalent intermolecular interactions using local properties. J Chem Inf Model 52:1061–1071. doi:10.1021/ci300095x 10.1021/ci300095x 22458324

[24] Clark T, Halik M, Hennemann M, Jäger CM (2013) Simulating “soft” electronic devices. In: Hicks MG, Kettner C (eds) Molecular engineering and control. Logos, Berlin, pp 137–150

[25] Etschel S, Waterloo A, Margraf JT, Amin AY, Hampel F, Jäger CM, Clark T, Halik M, Tykwinski RR (2013). An unsymmetrical pentacene derivative with ambipolar behavior in organic thin-film transistors. Chem Commun.

[26] Jäger CM, Schmaltz T, Novak M, Khassanov A, Vorobiev A, Hennemann M, Krause A, Dietrich H, Zahn D, Hirsch A, Halik M, Clark T (2013) Improving the charge transport in self-assembled monolayer field-effect transistors—from theory to devices. J Am Chem Soc 135:4893–4900. doi:10.1021/ja401320n 10.1021/ja401320n 23480792

[27] Schubert C, Margraf JT, Clark T, Guldi DM (2014) Molecular wires—impact of π-conjugation and implementation of molecular bottlenecks. Chem Soc Rev 44:988–998. doi:10.1039/c4cs00262h 10.1039/c4cs00262h 25316408

[28] Meyer B, Marx D (2003). Density-functional study of the structure and stability of ZnO surfaces. Phys Rev B.

[29] Kovacik R, Meyer B, Marx D (2007) F centers versus dimer vacancies on ZnO surfaces: characterization by STM and STS calculations. Angew Chem Int Ed 46:4894–4897. doi:10.1002/anie.200604399 10.1002/anie.200604399 17523200

[30] Horn AHC, Lin J-H, Clark T (2005) Multipole electrostatic model for MNDO-like techniques with minimal valence* spd*-basis sets. Theor Chem Acc 114:159–168; erratum: (2007) 117:461–465

[31] Atienza C, Martin N, Wielepolski M, Haworth N, Clark T, Guldi DM (2006) Tuning electron transfer through* p*-phenyleneethylene molecular wires. Chem Commun 30:3202–3204. doi: 10.1039/B603149H 10.1039/b603149h 17028743

[32] Bauer T (2015) Multi-Agenten-Simulation organischer Feldeffekttransistoren. Ph.D. thesis. Universität Erlangen-Nürnberg, Erlangen

[33] Ling MM, Reese C, Briseno AL, Bao Z (2007) Non-destructive probing of the anisotropy of field-effect mobility in the rubrene single crystal. Synth Met 157:257–260. doi:10.1016/j.synthmet.2007.02.004

[34] Reese C, Bao Z (2007) High-resolution measurement of the anisotropy of charge transport in single crystals. Adv Mater 19:4535–4538. doi:10.1002/adma.200701139

[35] Jämbeck JPM, Lyubartsev AP (2012) Derivation and systematic validation of a refined all-atom force field for phosphatidylcholine lipids. J Phys Chem B 116:3164–3179. doi:10.1021/jp212503e 10.1021/jp212503e PMC3320744 22352995

