# Supplementary material for: EMPIRE: a highly parallel semiempirical molecular orbital program: 2: periodic boundary conditions
Source: J Mol Model. 2015 May 17;21(6):144. doi: 10.1007/s00894-015-2692-3 (PMC4435633; doi:10.1007/s00894-015-2692-3)
Supplement: Supplementary file 1 — (DOCX 90 kb) [file 894_2015_2692_MOESM1_ESM.docx]

**Supporting Information for**

**EMPIRE: A highly parallel semiempirical molecular orbital program: 2: Periodic boundary conditions**

Johannes T. Margraf, Matthias Hennemann, Bernd Meyer, Timothy Clark*

Computer-Chemie-Centrum and Interdisciplinary Center for Molecular Materials, Friedrich-Alexander-Universität Erlangen-Nürnberg, Nägelsbachstraße 25, 91052 Erlangen, Germany

E-Mail: tim.clark@fau.de


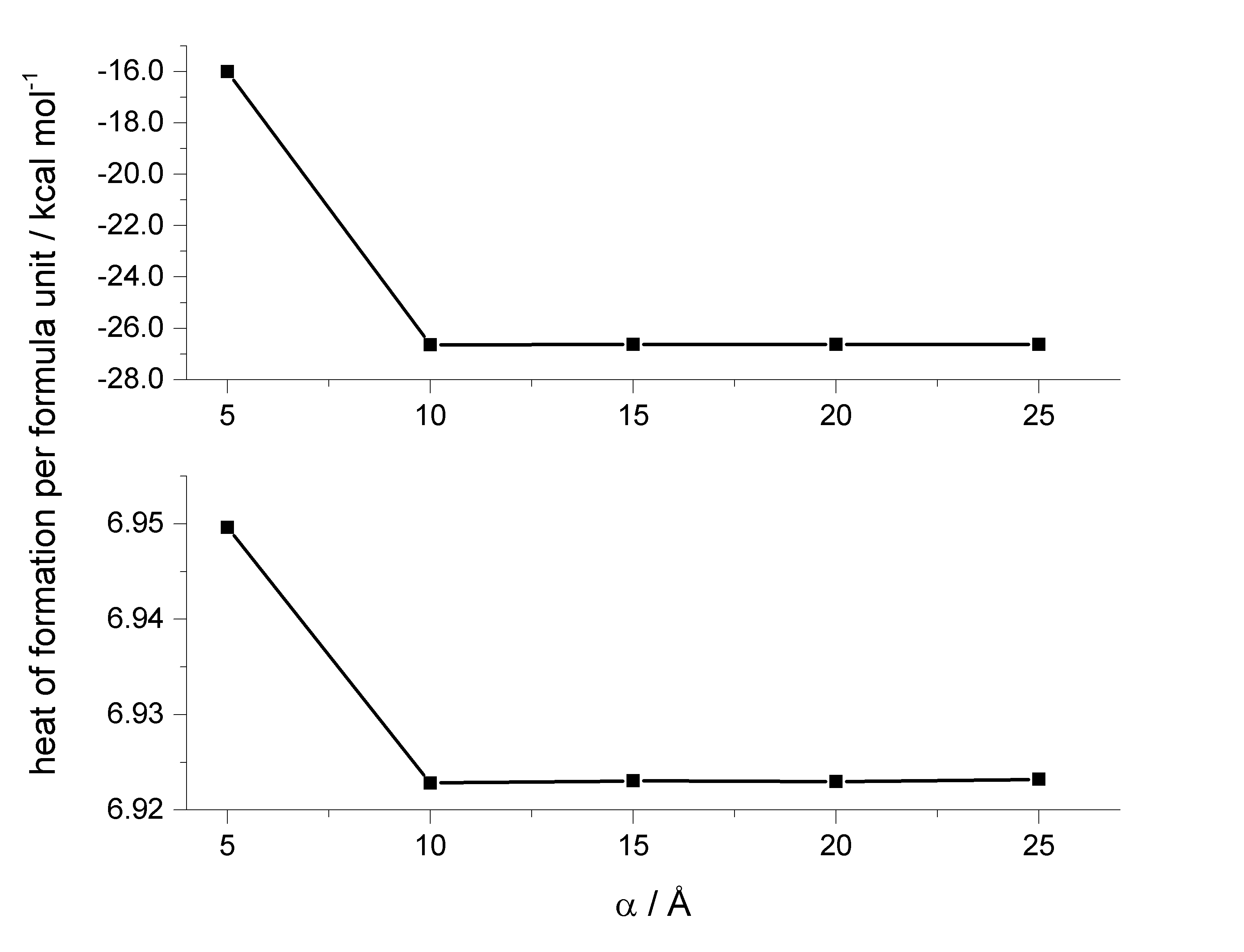


**Fig. S1: Convergece of the heat of formation with respect to the electrostatic screening parameter α for ZnO (top) and diamond (bottom).**


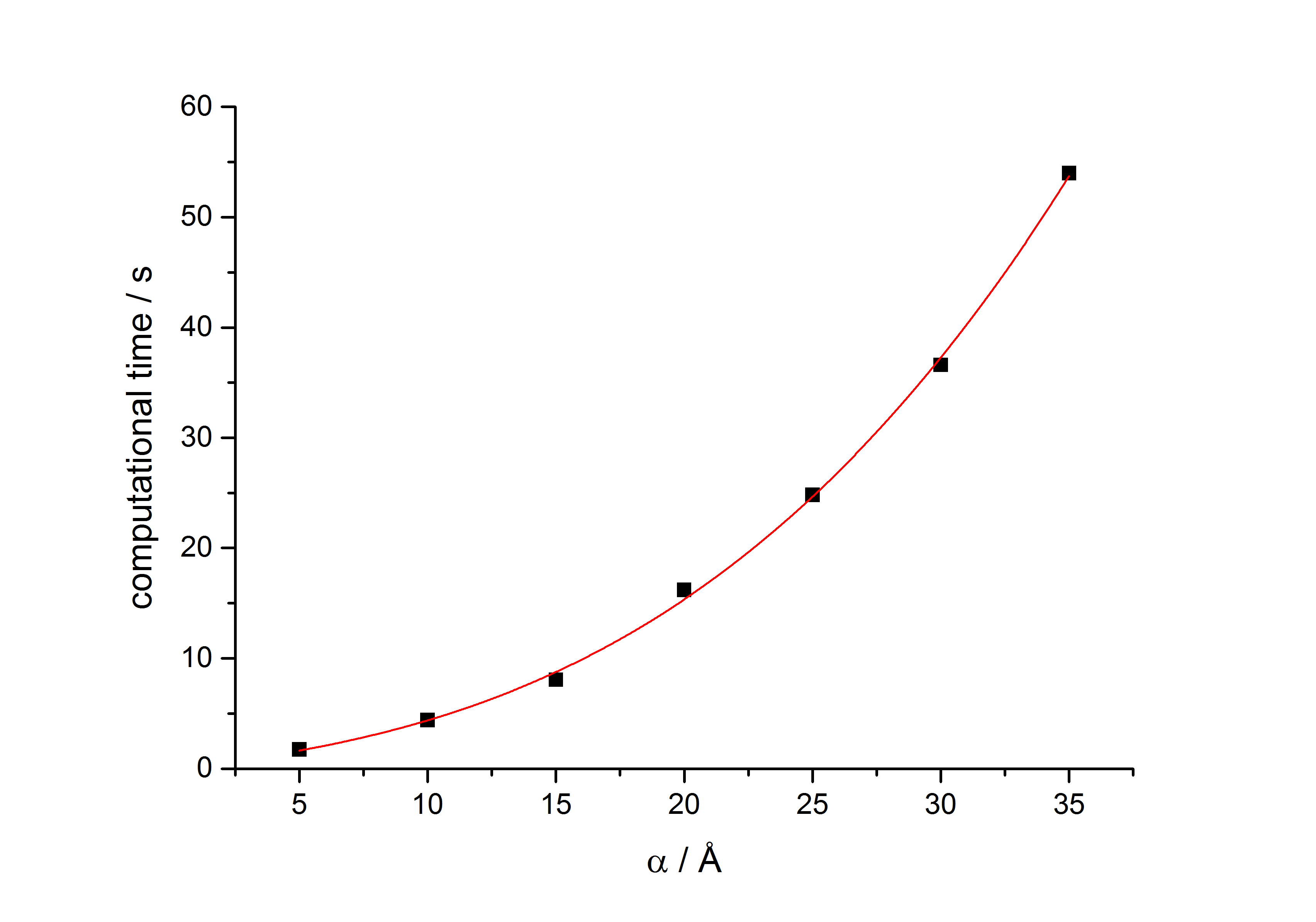


**Fig. S2: Computation time for a SCF calculation on a diamond C_216_ unit cell depending on the electrostatic screening parameter α. These calculations were performed with the single node version of EMPIRE using two quad-core 2.83 GHz Intel® Xenon® processors with 8 GB of memory. No hyperthreading was used.**
